# Alopecia areata and cardiovascular comorbidities: A cross-sectional analysis of the All of Us research program

**DOI:** 10.1016/j.jdin.2024.03.024

**Published:** 2024-04-08

**Authors:** Ambika Nohria, Jill T. Shah, Deesha Desai, Lina Alhanshali, Jenne Ingrassia, Alisa Femia, Michael Garshick, Jerry Shapiro, Kristen I. Lo Sicco

**Affiliations:** a The Ronald O Perelman Department of Dermatology, New York University Grossman School of Medicine, New York, New York; b University of Pittsburgh School of Medicine, Pittsburgh, Pennsylvania; c State University of New York System (SUNY) Downstate College of Medicine, Brooklyn, New York; d New York Medical College, Valhalla, New York

**Keywords:** alopecia, alopecia areata, comorbidity, cardiovascular disease, chronic kidney disease, hyperlipidemia, hypertension, obesity, valvular heart disease, atrial fibrillation, coronary artery disease, epidemiology, heart failure, myocardial infarction, stroke, type 2 diabetes, venous thromboembolism

*To the Editor:* Patients with alopecia areata (AA) are known to have an increased risk of comorbidities, such as autoimmune, atopic, and psychiatric diseases.^1^ Research has shown several inflammatory dermatologic conditions that are also associated with an increased risk of cardiovascular disease (CVD); however, the evidence for AA is conflicting.^2^^,^^3^ Our objective in the study was to evaluate the relationship between AA and CVD using the All of Us research program (AoU)—a National Institute of Health initiative aiming to collect health information from a diverse sample of >1 million US patients.

We performed a matched, cross-sectional analysis among AoU participants with >1 year of longitudinal electronic health record (EHR) data. We identified AA cases and controls with nearest neighbor propensity score matching by age, sex, race or ethnicity, and EHR duration. We compared demographics, clinical characteristics, indicators of socioeconomic status, and 12 traditional CV comorbidities and risk factors using Pearson’s χ^2^ test, Fisher’s exact test, unpaired *t* test, or Mann-Whitney U test. We corrected *P* values (Benjamini-Hochberg procedure; false discovery rate: 5%). Multivariable conditional logistic regression was used with the inclusion of comorbidities and potential confounders with *P* value of <.1 in univariable analysis.

We identified 995 AA cases, 74.4% female with a mean age of 56 years (IQR, 41-66 years) and 3980 matched controls. Compared with controls, patients with AA were significantly more likely to have an annual household income of >35K, completed high school, be actively employed, and endorse no history of smoking (Table I). In multivariable analysis adjusting for potential confounders such as income and smoking history, patients with AA had a significantly higher odds ratio (OR) for the development of chronic kidney disease (CKD) (OR, 1.3; 95% CI, 1.03-1.6; *P* = .025) and hyperlipidemia (OR, 1.3; 95% CI, 1.1-1.5; *P* = .002). Factors not significantly associated in univariable or multivariable analyses include atrial fibrillation, coronary artery disease, congestive heart failure, hypertension, myocardial infarction, obesity, stroke/transient ischemia attack, type 2 diabetes mellitus, valvular heart disease, and venous thromboembolism (Tables I and II).

**Table I:** Demographic and clinical characteristics and association of comorbid conditions in patients with alopecia areata cases vs matched controls

Characteristic	Alopecia areata cases	Matched controls	*P*	Adjusted *P*∗
*N*	995	3980		
Age, y; mean (range)	56 (41-66)	56 (40-67)	.832	
Sex at birth, *N* (%)			.667	
Female	740 (74.4)	3007 (75.6)		
Male	230 (23.1)	868 (21.8)		
Other/unknown	25 (2.5)	105 (2.6)		
Race/ethnicity, *N* (%)			.487	
Asian	43 (4.3)	184 (4.6)		
Black/African American	214 (21.5)	930 (23.4)		
Hispanic/Latino	242 (24.3)	937 (23.5)		
White	412 (41.4)	1554 (39.0)		
Other/more than one/unknown	84 (8.4)	375 (9.4)		
Duration of EHR, y; median (IQR)	16 (9-24)	15 (8-23)	.156	
Annual household income, *N* (%)			<.001	
>35K	502 (50.5)	1721 (43.2)		
<35K	282 (28.3)	1360 (34.2)		
Unknown	211 (21.2)	899 (22.6)		
Education, *N* (%)			.004	
High school completed	884 (88.8)	3379 (84.9)		
Less than high school	77 (7.7)	441 (11.1)		
Unknown	34 (3.4)	160 (4.0)		
Employment status, *N* (%)			.003	
Employed for wages/self-employed	519 (52.2)	1845 (46.4)		
Not employed for wages	431 (43.3)	1961 (49.3)		
Unknown	45 (4.5)	174 (4.4)		
Marital status, *N* (%)			.321	
Divorced, separated, or widowed	254 (25.5)	943 (23.7)		
Married or living with partner	468 (47.0)	1858 (46.7)		
Never married	227 (22.8)	1010 (25.4)		
Unknown	46 (4.6)	169 (4.2)		
Smoking status†, *N* (%)			<.001	
Ever smoker	310 (31.2)	1491 (37.5)		
Not ever smoker	656 (65.9)	2348 (59.0)		
Unknown	29 (2.9)	141 (3.5)		
Atrial fibrillation, *N* (%)	73 (7.3)	256 (6.4)	.304	.456
Chronic kidney disease, *N* (%)	135 (13.6)	412 (10.4)	.004	.024
Coronary artery disease, *N* (%)	139 (13.9)	543 (13.6)	.788	.788
Congestive heart failure, *N* (%)	65 (6.5)	289 (7.3)	.424	.565
Hyperlipidemia, *N* (%)	546 (54.9)	1875 (47.1)	<.001	<.001
Hypertension, *N* (%)	543 (54.6)	2008 (50.5)	.02	.06
Myocardial infarction, *N* (%)	58 (5.8)	215 (5.4)	.597	.651
Obesity, *N* (%)	413 (41.1)	1472 (37.0)	.008	.032
Stroke /transient ischemic attack, *N* (%)	41 (4.1)	137 (3.4)	.3	.514
Type 2 diabetes mellitus, *N* (%)	250 (25.1)	965 (24.2)	.563	.676
Valvular heart disease, *N* (%)	78 (7.8)	253 (6.4)	.093	.223
Venous thromboembolism, *N* (%)	76 (7.6)	255 (6.4)	.163	.326

*EHR*, Electronic health record.

∗ *P* values are adjusted to account for the false discovery rate (5%) using the Benjamini-Hochberg procedure.

† Defined as having smoked at least 100 cigarettes (5 packs) in one’s lifetime.

**Table II:** Multivariable conditional logistic regression model∗

Condition	Multivariable *P*	Multivariable odds ratio (95% CI)
Chronic kidney disease	.025	1.3 (1.03-1.6)
Hyperlipidemia	.002	1.3 (1.1-1.5)
Hypertension	.423	1.1 (0.9-1.3)
Obesity	.068	1.2 (0.98-1.3)

∗ One model was created with all conditions and potential confounders from Table I with *P* or adjusted *P* <.1. This model adjusts for smoking status, income, education status, and employment status. Variables with missing data were treated as a separate category in regression models.

Although the psychosocial impact of AA is well-established, the impact on internal disease, specifically CVD is not fully understood. Our findings showed that patients with AA were not directly at increased risk for adverse cardiovascular events including coronary artery disease, myocardial infarction, stroke, and venous thromboembolism. However, there was a significant association with CKD and hyperlipidemia, which may place patients at an elevated risk of CVD.^4^ This study is limited by sample size, potential inaccuracies in EHR-based diagnoses, and potential inaccuracies in self-reported race and ethnicity data in AoU. Our cohort was also predominantly female with a mean age of ≤65 years, which may be a potential confounder as CVD is more common in older males. Although we did not find a direct association with cardiovascular events, the associations with hyperlipidemia and CKD still have important implications for patient counseling, particularly as Janus Kinase Inhibitors are increasingly used to treat AA. The Janus Kinase Inhibitors are currently the only US Food and Drug Administration-approved treatment for severe AA; however, this class of medications may confer further cardiovascular risk.^5^ Further investigation of CVD is necessary and increased monitoring may be warranted for patients with AA.

## Conflicts of interest

Dr Lo Sicco has been an investigator for Regen Lab and is an investigator for Pfizer. Dr Lo Sicco is a consultant for Pfizer and Aquis. Dr Shapiro is an investigator and consultant for Pfizer and is a consultant for Lilly. Dr Femia receives royalties from UpToDate; she reports consulting fees from Octagon Therapeutics and Timber Pharmaceuticals. Dr Garshick reports consulting fees from AbbVie and Horizon Therapeutics. The authors have no conflicts of interest to declare.
